# A real-world, triple-cohort study on the effectiveness and safety of telitacicept in immunosuppressant-naïve and refractory lupus nephritis

**DOI:** 10.3389/fimmu.2026.1748931

**Published:** 2026-09-16

**Authors:** Suren Lai, Shanshan Li, Jiaying Cai, Lingyun Sun, Huasang Huang, Jie Wang, Jinhai Chen, Shizhong Shen

**Affiliations:** 1 Department of Nephrology, The First Affiliated Hospital of Quanzhou, Fujian Medical University, Quanzhou, China; 2 Department of Nephrology, Wenling First People’s Hospital, Wenling, China; 3 Department of Nephrology, The Second Affiliated Hospital of Fujian Medical University, Quanzhou, China

**Keywords:** APRIL, BAFF, dual-target therapy, lupus nephritis, real-world study, refractory, telitacicept, treatment-naïve

## Abstract

**Objective:**

To compare the efficacy and safety of telitacicept as add-on therapy versus standard of care (SOC) alone in immunosuppressant (IS)-naïve patients with lupus nephritis (LN) and in patients with refractory LN.

**Methods:**

In this bicentric, real-world, retrospective, triple-cohort study, we enrolled IS-naïve LN patients receiving add-on telitacicept (Cohort 1), IS-naïve LN patients receiving SOC alone (Cohort 2), and refractory LN patients receiving add-on telitacicept (Cohort 3). The primary endpoint was time to complete remission (CR). Secondary endpoints included renal remission rates at weeks 24 and 48, changes in serological and renal function parameters, glucocorticoid reduction, and safety. We assessed time to CR using the Kaplan-Meier (KM) method, Cox regression, and restricted mean event time (RMET) analysis if KM curves crossed early. Least squares mean (LSM) changes were estimated.

**Results:**

We enrolled 55 eligible patients. Patients in Cohorts 1 (N = 16) and 3 (N = 17) received telitacicept 160 mg subcutaneously weekly in addition to SOC therapy; those in Cohort 2 (N = 22) received SOC alone. SOC regimens were predominantly mycophenolate mofetil-based. In IS-naïve patients, Cohort 1 achieved CR numerically faster than Cohort 2 (HR = 1.268, 95% CI: 0.567–2.834, P = 0.578; RMET difference: −3.96 weeks, 95% CI: −17.13 to 9.21, P = 0.556). Among patients receiving telitacicept, Cohort 1 showed numerically higher CR (75.0% vs 64.7%) and TR rates (93.8% vs 76.5%) than Cohort 3, with numerically faster CR achievement (HR = 1.364, 95% CI: 0.600–3.102; RMET difference: −4.89 weeks; 95% CI: −18.50 to 8.73). Both telitacicept-treated cohorts showed substantial laboratory improvements, with Cohort 1 demonstrating the greatest eGFR improvement (+23.4 mL/min/1.73m²). A glucocorticoid-sparing effect was observed. Infections were the most common TEAEs; all were mild to moderate.

**Conclusion:**

Telitacicept demonstrated favorable efficacy and manageable safety in both IS-naïve and refractory LN patients. In the treatment-naïve setting, telitacicept achieved CR more quickly than SOC. Faster, higher responses in IS-naïve versus refractory patients suggest a potential benefit of early intervention. These findings support early use of telitacicept and underscore the need for larger randomized controlled trials to confirm the benefits of early dual-target B-cell therapy in LN.

## 1 Introduction

Lupus nephritis (LN), a serious and common complication of systemic lupus erythematosus (SLE), affects 40%-60% of patients and remains a major cause of morbidity and mortality. Despite standard-of-care treatment with hydroxychloroquine (HCQ), glucocorticoids (GCs), and immunosuppressants (IS) such as mycophenolate mofetil or cyclophosphamide, or calcineurin inhibitors such as voclosporin, many patients respond inadequately or are intolerant, leading to refractory LN. Additionally, relapse rates stay high (33%-40%) among initial responders, and long-term use of high-dose GCs and IS contributes to significant toxicity (1–4).

The crucial role of B cells in SLE development has led to the creation of biologic agents targeting B-cell pathways. Rituximab, an anti-CD20 monoclonal antibody, showed limited success in controlled LN trials, underscoring the difficulties in B-cell targeting (5). Conversely, belimumab, a monoclonal antibody that blocks the B-cell survival factor BAFF, has proven effective in LN, confirming this pathway as a therapeutic target (6). However, the concurrent APRIL pathway, which is vital for the survival of long-lived plasma cells, the main producers of autoantibodies, is not affected by belimumab (7).

Telitacicept offers a novel therapeutic approach as a recombinant TACI-Fc fusion protein that simultaneously neutralizes both BAFF and APRIL (8). This dual-target mechanism allows for more extensive suppression of B-lineage cells, including long-lived plasma cells, potentially providing greater efficacy than agents that target only one molecule (9). Although telitacicept has demonstrated promise in SLE clinical trials and gained regulatory approval in China, comprehensive real-world data specifically in LN populations, especially when comparing immunosuppressants (IS)-naïve and refractory patients, remains limited (10).

This bicentric, real-world, retrospective, triple-cohort study was designed to address two comparative aims: (1) to compare telitacicept add-on to SOC with SOC alone in the treatment of IS-naïve LN patients; and (2) to compare telitacicept in the treatment of IS-naïve LN versus refractory LN patients.

## 2 Materials and methods

### 2.1 Study design and participants

This bicentric, retrospective, triple-cohort study was conducted at the Departments of Nephrology at the First and Second Affiliated Hospitals of Fujian Medical University. The inclusion criteria for all participants were: (1) age ≥18 years at the time of confirmed LN diagnosis and meeting the 2019 EULAR/ACR classification criteria for SLE (11); (2) biopsy-confirmed LN with active lesions; (3) serum positivity for antinuclear antibodies (ANAs) and/or anti–double-stranded DNA (anti-dsDNA) antibodies at the time of the first confirmed LN diagnosis; (4) available Kidney-biopsy LN classification according to the International Society of Nephrology/Renal Pathology Society (ISN/RPS) (12); (5) a ratio of urinary protein to creatinine (UPCR) > 1.0 g/g (100 mg/mmol) based on a 24-hour urine sample collection; (6) at least 48 weeks of follow-up. Exclusion criteria included: (1) dialysis within 1 year; (2) severe infections or significant cardiopulmonary issues; (3) treatment with rituximab or other B-cell-targeted biologics at any time prior to initiating telitacicept. We screened eligible patients through electronic medical record systems (EMR) at both centers. Eligible patients were included and divided into three cohorts: (1) IS-naïve LN patients who had received only GCs or HCQ prior to receiving telitacicept as an add-on to standard-of-care (SOC) therapy (IS-naïve + Telitacicept, Cohort 1); (2) IS-naïve LN patients who had received only GCs or HCQ prior to receiving SOC alone (IS-naïve + SOC, Cohort 2); (3) Refractory LN patients, defined as those who failed to achieve at least a partial renal response (≥50% reduction in UPCR) despite an appropriate 6-month course of SOC therapy (IS, including MMF, cyclophosphamide, etc.; or calcineurin inhibitors) prior to receiving telitacicept as an add-on to SOC therapy (Refractory + Telitacicept, Cohort 3). **Supplementary Table A1** provides a detailed summary of prior IS exposure for this cohort.

### 2.2 Study objectives

This study was designed to achieve two primary objectives: (1) to compare the efficacy and safety of telitacicept add-on to SOC with SOC alone in IS-naïve LN patients (Cohort 1 vs Cohort 2); and (2) to compare the efficacy and safety of telitacicept in IS-naïve LN versus refractory LN patients (Cohort 1 vs Cohort 3).

The secondary objective was to assess the safety profile of telitacicept across all treatment cohorts.

### 2.3 Data collection and outcomes

Clinical and laboratory data were systematically collected at baseline (just before the first dose of telitacicept) and then at 4, 12, 24, and 48 weeks from the electronic medical record systems in both centers. The primary efficacy endpoint was the time to complete remission (CR).

CR was defined as a urine protein-to-creatinine ratio (UPCR) <0.5 g/g with normal or near-normal estimated glomerular filtration rate (eGFR) of ≥60 mL/min/1.73 m² or no more than a 20% decline from baseline; partial remission (PR) as ≥50% reduction in UPCR with stable renal function; and total remission (TR) as either complete or partial remission.

Key secondary efficacy endpoints were the renal remission rate, including CR and TR rates, at weeks 24 and 48. Additionally, changes from baseline at weeks 24 and 48 were measured for UPCR, eGFR, complement levels (C3, C4), immunoglobulin levels (IgG, IgA, IgM), hemoglobin (Hb), blood platelet count (BPC), and glucocorticoid dose, standardized to methylprednisolone. Safety was evaluated by assessing the incidence and characteristics of adverse events throughout the study period.

### 2.4 Statistical analysis

Statistical analyses were conducted in R (version 4.5.0). Continuous variables were described as mean ± standard deviation (SD) or median and 25–75 interquartile range (IQR). Categorical variables are reported as proportions. Within-cohort changes from baseline were assessed using paired t-tests or Wilcoxon signed-rank tests, as appropriate. Between-cohort comparisons were performed using Wilcoxon rank-sum tests. For time-to-event analysis, we used Kaplan-Meier (KM) analysis and Cox proportional hazards regression to estimate the hazard ratio (HR) and 95% confidence interval (CI) for complete remission. If KM curves crossed early, we performed restricted mean event time (RMET) analysis to estimate the time to CR instead of KM analysis. Least squares mean (LSM) changes from baseline to Weeks 24 and 48 were estimated using analysis of covariance (ANCOVA), with baseline values included as covariates. This approach adjusts for potential baseline imbalances and provides more robust estimates of treatment effects. The ANCOVA models included treatment cohort as the independent variable and baseline parameter as the covariate. We calculated LSM changes with 95% confidence intervals for each cohort. A P-value of less than 0.05 was considered statistically significant for all analyses.

## 3 Results

### 3.1 Patients characteristics

Between March 2021 and May 2024, we retrospectively enrolled 55 patients with active lupus nephritis and divided them into three cohorts: 16 in Cohort 1, 22 in Cohort 2, and 17 in Cohort 3. Cohort 1 and Cohort 3 received telitacicept at 160 mg weekly via subcutaneous injection as an add-on to SOC treatment; Cohort 2 received SOC treatment only. Glucocorticoids, with doses standardized to methylprednisolone, and IS used in SOC regimens are summarized in **Table 1** and **Supplementary Table A2**. Mycophenolate mofetil (MMF) was the most common IS across all cohorts, administered to all in Cohort 1 (16/16, 100.0%), 59.1% in Cohort 2 (13/22), and 88.2% in Cohort 3 (15/17). Cyclophosphamide was used rarely, with one patient each in Cohorts 2 (4.5%) and 3 (5.9%). Tacrolimus monotherapy was only in Cohort 2 (2/22, 9.1%), as was leflunomide monotherapy (3/22, 13.6%). Dual therapy with MMF plus tacrolimus was in two patients in Cohort 2 (9.1%) and one in Cohort 3 (5.9%). One patient in Cohort 2 (4.5%) received steroids alone. Overall, the regimens reflected real-world practice, with most patients on MMF-based therapy.

**Table 1:** Baseline characteristics.

Characteristic	A. Cohort 1 (N = 16)	B. Cohort 2 (N = 22)	C. Cohort 3 (N = 17)	p-values	
				A vs B	A vs C
Gender, n (%)				0.234	1.000
Female	14 (87.5)	16 (72.7)	15 (88.2)		
Male	2 (12.5)	6 (27.3)	2 (11.8)		
Age (years)	31.9 ± 9.4	42.7 ± 21.0	37.0 ± 12.1	0.085	0.367
Activity Index, median (IQR)	10 (6.8, 11)	6 (5, 10.8)	10 (9, 12)	0.136	0.675
Chronicity Index, median (IQR)	1 (1, 2.2)	1 (0, 2)	3 (2, 3)	0.459	0.029
SLEDAI-2K score, median (IQR)	13 (10, 17)	10 (10, 12)	10 (10, 14)	0.042	0.263
eGFR (mL/min/1.73m²)	86.2 ± 42.5	88.0 ± 48.4	58.5 ± 35.2	0.916	0.050
UPCR (mg/mmol)	445.4 ± 269.0	428.7 ± 282.6	498.5 ± 580.5	0.856	0.460
ESR, mm/h)	65.2 ± 32.5	65.3 ± 37.8	43.7 ± 37.5	0.849	0.058
IgA (g/L)	3.25 ± 0.87	2.76 ± 1.07	2.15 ± 0.92	0.147	0.004
IgM (g/L)	1.26 ± 0.8	1.32 ± 0.77	1.13 ± 0.94	0.701	0.288
IgG (g/L)	10.58 ± 5.39	11.29 ± 5.08	11.11 ± 4.52	0.929	0.957
Complement C3 (g/L)	0.38 ± 0.27	0.49 ± 0.20	0.58 ± 0.31	0.185	0.050
Complement C4 (g/L)	0.07 ± 0.06	0.11 ± 0.06	0.14 ± 0.10	0.066	0.020
ISN/RPS LN class, n (%)				0.490	0.162
Class III	0 (0%)	1 (4.5%)	3 (17.6%)		
Class IV	5 (31.2%)	4 (18.2%)	4 (23.5%)		
Class V	3 (18.8%)	6 (27.3%)	0 (0%)		
Class III/V	2 (12.5%)	6 (27.3%)	3 (17.6%)		
Class IV/V	6 (37.5%)	4 (18.2%)	5 (29.4%)		
Class III/IV	0 (0%)	0 (0%)	0 (0%)		
ANA positive	16/16 (100%)	20/22 (90.9%)	16/17 (94.1%)	0.499	1.000
Anti-dsDNA positive	6/16 (37.5%)	7/22 (31.8%)	6/17 (35.3%)	0.742	1.000
Glucocorticoid (mg/d)	37.6 ± 12.5	42.3 ± 8.8	19.2 ± 16.5	0.184	0.005

Data were presented as mean ± SD unless otherwise specified. UPCR, urine protein-to-creatinine ratio. LN, lupus nephritis. GC, glucocorticoids. eGFR, estimated glomerular filtration rate; Anti-dsDNA, anti-double-stranded DNA; SLEDAI-2K, Systemic Lupus Erythematosus Disease Activity Index 2000; MMF, mycophenolate mofetil; IQR, interquartile range. Cohort 1, IS-naïve + Telitacicept. Cohort 2, IS-naïve + SOC. Cohort 3, Refractory+Telitacicept. Glucocorticoid dose had been standardized to methylprednisolone.

**Table 1** and **Supplementary Table A3** summarize the baseline characteristics. Between Cohort 1 and Cohort 2, the SLEDAI-2K score was slightly higher in Cohort 1 (13 vs 10, P = 0.042). All other baseline variables were well balanced, with all P values > 0.05 (age: P = 0.085; gender: P = 0.234; eGFR: P = 0.916; UPCR: P = 0.856; C3: P = 0.185; C4: P = 0.066; Activity index: P = 0.136; Chronic index: P = 0.459; glucocorticoid: P = 0.184), indicating that the two cohorts were comparable at baseline. For the comparison between Cohort 1 and Cohort 3, Cohort 3 was well balanced for age and gender. As expected, Cohort 3 had significantly lower eGFR (58.5 vs 86.2 mL/min/1.73m², P = 0.050), higher complement C4 (0.14 vs 0.07 g/L, P = 0.020), higher Chronic index than Cohort 1 (median: 3 vs 1, P = 0.029), and lower baseline glucocorticoid doses (19.2 vs 37.6 mg/d, P = 0.005), reflecting the expected clinical differences between treatment-naïve and refractory populations.

### 3.2 Renal remission

Time-to-CR analysis indicated a numerical advantage for Cohort 1 over Cohorts 2 and 3, though the differences were not statistically significant. The KM curves crossed early. In the comparison between Cohort 1 and Cohort 2, Cox regression showed a hazard ratio (HR) of 1.268 (95% CI: 0.567–2.834, P = 0.578). RMET analysis at τ = 55 weeks showed that Cohort 1 achieved CR on average 3.96 weeks earlier than Cohort 2 (RMET difference: −3.96 weeks; 95% CI: −17.13 to 9.21; P = 0.556) (**Figures 1C, D**). For Cohort 1 vs Cohort 3, Cox regression showed an HR of 1.364 (95% CI: 0.600–3.102; Log-rank P = 0.459). RMET analysis showed that Cohort 1 achieved CR on average 4.89 weeks earlier than Cohort 3 (RMET difference: −4.89 weeks; 95% CI: −18.50 to 8.73; P = 0.482) (**Figures 1C, E**).

**Figure 1:**
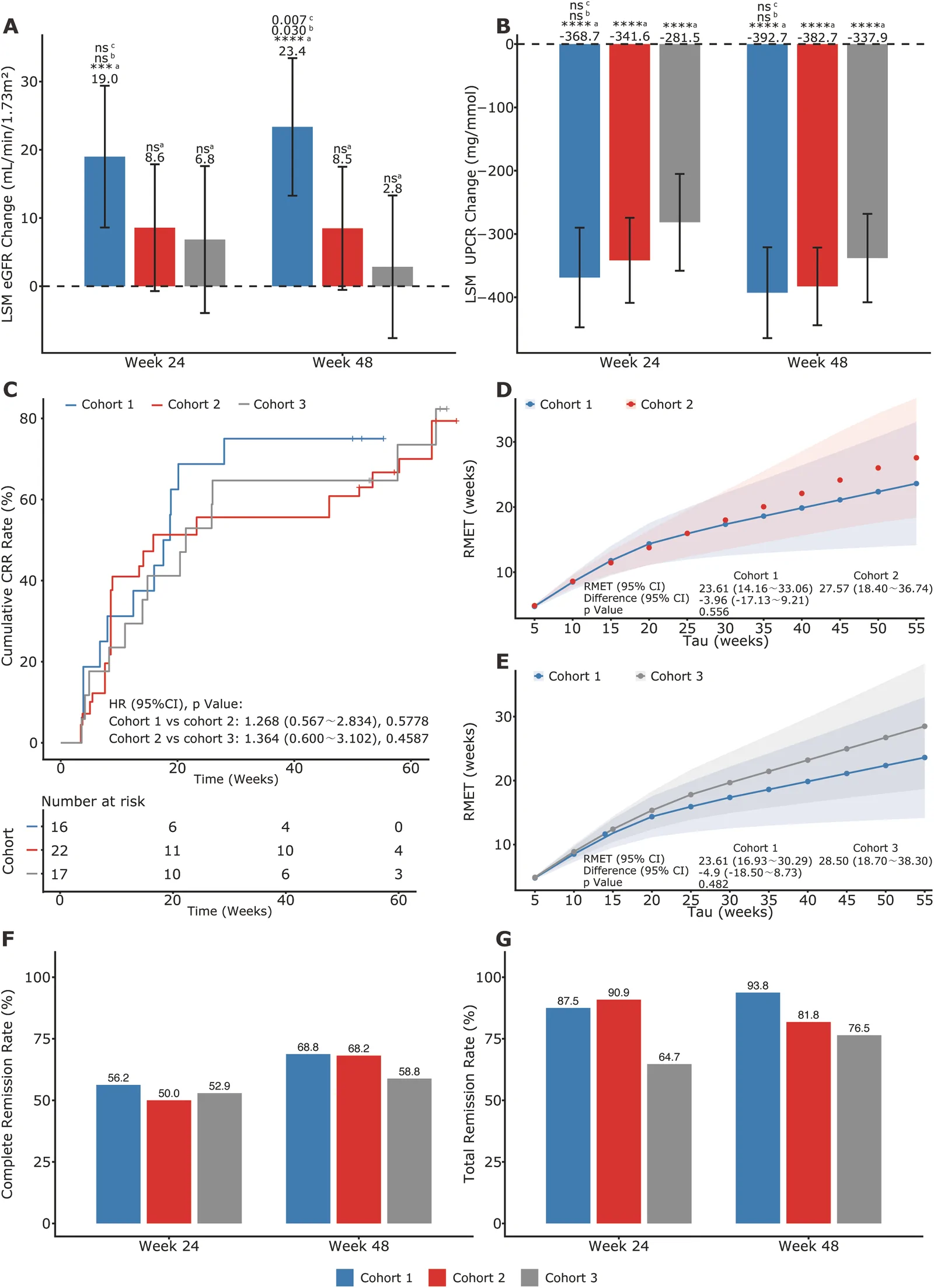
Renal efficacy outcomes in treatment-naïve and refractory lupus nephritis patients treated with telitacicept. **(A)** Longitudinal changes in urine protein-to-creatinine ratio (UPCR). **(B)** Changes in estimated glomerular filtration rate (eGFR). **(C)** Kaplan-Meier curves for time to complete renal remission. **(D)** RMET for IS-naïve + Telitacicept cohort vs IS-naïve + SOC cohort. **(E)** RMET for IS-naïve + Telitacicept cohort vs Refractory+Telitacicept cohort. **(F)** Complete remission rates at weeks 24 and 48. **(G)** Total remission rates at weeks 24 and 48. CRR, complete renal remission. HR, Hazard ratio. CI, confidence interval. Cohort 1, IS-naïve + Telitacicept (n = 16). Cohort 2, IS-naïve + SOC (n = 22). Cohort 3, Refractory+Telitacicept (n = 17). RMET, restricted mean event time. **** P < 0.0001, *** P < 0.001, ** P < 0.01, * P < 0.05, ns, not significant. a, p-value within the cohort. b, p-value between cohorts 1 and 2. c, p-value between cohorts 1 and 3.

CR rates improved steadily over the 48-week study period across the three cohorts. Cohort 1 had slightly higher CR rates at weeks 24 (56.2%) and 48 (68.8%), and these trends were consistent with TR rates, except that Cohort 2 showed a slightly higher TR rate (90.9%) at week 24 (**Figures 1F, G**). No statistically significant differences were observed between Cohorts 1 and 2 or 3 in CR or TR rates.

### 3.3 Changes from baseline of laboratory parameters

**Supplementary Table A4**; **Table 2** and **Figures 1**, **2** present least squares mean (LSM) changes from baseline to Weeks 24 and 48 for key laboratory parameters. Cohort 1 showed the greatest LSM improvement in eGFR from baseline to Week 48 (+23.4 mL/min/1.73m², 95% CI: 13.3–33.4), compared with Cohort 2 (+8.5 mL/min/1.73m², 95% CI: −0.5 to 17.5) and Cohort 3 (+2.8 mL/min/1.73m², 95% CI: −7.6 to 13.3). Between-cohort differences were statistically significant between Cohort 1 and Cohort 2 (P = 0.03) and between Cohort 1 and Cohort 3 (P = 0.007) (**Supplementary Table A4**; **Table 2**; **Figure 1A**). All three cohorts showed substantial reductions in UPCR, with LSM changes of −392.7 mg/mmol (95% CI: −464.5 to −320.9) in Cohort 1, −382.7 mg/mmol (95% CI: −444.0 to −321.4) in Cohort 2, and −337.9 mg/mmol (95% CI: −407.7 to −268.1) in Cohort 3. Within-cohort P values were < 0.0001. No significant between-cohort differences were observed (Cohort 1 vs Cohort 2: P = 0.83; Cohort 1 vs Cohort 3: P = 0.28) (**Supplementary Tables A4**; **Table 2**; **Figure 1B**).

**Table 2:** LSM changes in laboratory parameters from baseline to week 48.

Measures	LSM change from baseline (95% CI) ^†^			p value	
	A. Cohort 1 (N = 16)	B. Cohort 2 (N = 22)	C. Cohort 3 (n=17)	A vs B	A vs C
eGFR (mL/min/1.73m²)	23.4 (13.3, 33.4) ^****^	8.5 (-0.5, 17.5) ^ns^	2.8 (-7.6, 13.3) ^ns^	0.030	0.007
UPCR (mg/mmol)	-392.71 (-464.5, -320.92) ^****^	-382.68 (-444, -321.37) ^****^	-337.92 (-407.73, -268.11) ^****^	0.83	0.28
C3 (g/L)	0.40 (0.3, 0.5) ^****^	0.31 (0.24, 0.39) ^****^	0.35 (0.26, 0.43) ^****^	0.16	0.44
C4 (g/L)	0.07 (0.03, 0.11) **	0.12 (0.09, 0.16) ****	0.11 (0.07, 0.15) ^****^	0.05	0.20
IgA (g/L)	-1.22 (-1.91, -0.54) ^***^	-0.73 (-1.24, -0.22) ^**^	-1.09 (-1.69, -0.48) ^***^	0.25	0.77
IgG (g/L)	-2.13 (-3.75, -0.52) ^*^	-0.92 (-2.16, 0.32) ^ns^	-2.87 (-4.28, -1.46) ^***^	0.24	0.49
IgM (g/L)	-0.81 (-1.02, -0.61) ^****^	-0.41 (-0.57, -0.26) ^****^	-0.82 (-1, -0.64) ^****^	0.002	0.980
Serum Albumin (g/L)	15.64 (13.78, 17.51) ^****^	12.08 (10.5, 13.65) ^****^	12.76 (10.92, 14.61) ^****^	0.005	0.040
Hb (g/L)	28.59 (22.83, 34.34) ^****^	14.83 (9.86, 19.8) ^****^	13.14 (7.55, 18.72) ^****^	0.0007	0.0003
BPC (×10^9^/L)	54.39 (27.52, 81.25) ^***^	27.61 (5.15, 50.08) ^*^	37.37 (11.41, 63.33) ^**^	0.13	0.37

LSM, least squares mean. C3, C4, complement C3, C4. Ig, immunoglobulin. BPC, blood platelet count. ^†^Indicated p-values within the cohort for LSM changes in measures from baseline: ****P < 0.0001, ***P < 0.001, **P < 0.01, *P < 0.05, ns, not significant. Cohort 1, IS-naïve + Telitacicept. Cohort 2, IS-naïve + SOC. Cohort 3, Refractory+Telitacicept.

**Figure 2:**
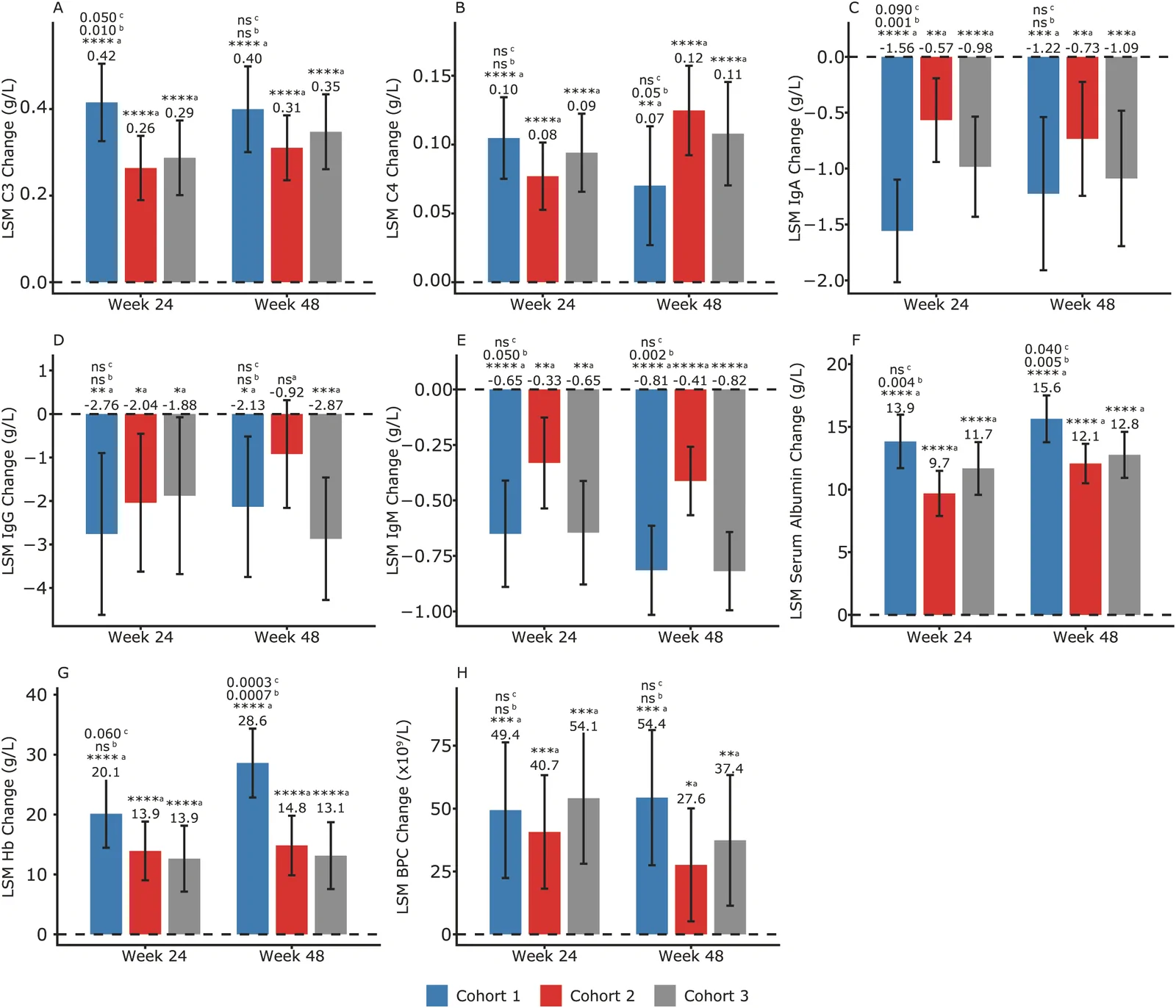
Comprehensive response to telitacicept treatment: recovery of complement levels **(A, B)**; reduction in immunoglobulins **(C–E)**; improvement in serum albumin levels **(F)**; enhancement of hematological profile **(G, H)**. C3, C4, Complement C3, C4. Ig, Immunoglobulin. Hb, hemoglobin. BPC, blood platelet count. Repeated measures ANOVA was performed. Data are shown as mean ± 95% confidence interval. Cohort 1, IS-naïve + Telitacicept. Cohort 2, IS-naïve + SOC. Cohort 3, Refractory+Telitacicept. **** P < 0.0001, *** P < 0.001, ** P < 0.01, * P < 0.05, ns, not significant. a, p-value within the cohort. b, p-value between cohorts 1 and 2. c, p-value between cohorts 1 and 3.

Immunological profiling showed robust serological responses across all cohorts, with significant increases in complement C3 and C4 levels and significant reductions in immunoglobulin levels (IgA, IgG, IgM) (**Supplementary Tables A4**; **Table 2**; **Figures 2A–E**). Serum albumin levels increased significantly across all cohorts (**Supplementary Tables A4**; **Table 2**; **Figure 2F**). Hematological parameters (hemoglobin and platelet counts) also improved significantly (**Supplementary Tables A4**; **Table 2**; **Figures 2G, H**).

A glucocorticoid-sparing effect was observed across all cohorts (**Table 3**). In Cohort 1, glucocorticoid dose decreased from 37.6 ± 12.5 mg/d at baseline to 7.7 ± 4.6 mg/d at Week 24 (P < 0.001) and to 4.2 ± 3.2 mg/d at Week 48 (within-cohort p-value < 0.001). Cohort 2 showed reductions from 38.7 ± 10.9 mg/d to 10.9 ± 4.8 mg/d (within-cohort p-value < 0.0001) at Week 24 and to 6.8 ± 8.1 mg/d (within-cohort p-value < 0.0001) at Week 48. Cohort 3 showed reductions from 19.2 ± 16.5 mg/d to 5.4 ± 7.2 mg/d (within-cohort p-value < 0.01) at Week 24 and to 3.3 ± 3.8 mg/d (within-cohort p-value < 0.001) at Week 48. Between-cohort comparisons showed that Cohort 1 had lower glucocorticoid doses than Cohort 2 at Week 24 (p = 0.03). Complete glucocorticoid discontinuation by Week 48 was achieved in 4 of 16 patients (25.0%) in Cohort 1, 4 of 22 patients (18.2%) in Cohort 2, and 8 of 17 patients (47.1%) in Cohort 3.

**Table 3:** Glucocorticoid dose over time.

Time point	Glucocorticoid dose (mg/d) ^†^			p value	
	A. Cohort 1 (N = 16)	B. Cohort 2 (N = 22)	C. Cohort 3 (N = 17)	A vs B	A vs C
Baseline	37.6 ± 12.5	38.7 ± 10.9	19.2 ± 16.5	ns	0.005
Week 24	7.7 ± 4.6 ^***^	10.9 ± 4.8 ^****^	5.4 ± 7.2 ^**^	0.03	ns
Week 48	4.2 ± 3.2 ^***^	6.8 ± 8.1 ^****^	3.3 ± 3.8 ^***^	ns	Ns
Complete withdrawal, n (%)	4 (25.0%)	4 (18.2%)	8 (47.1%)	–	–

Data are presented as mean ± SD. ^†^p-values within the cohort compared to baseline: ****P < 0.0001, ***P < 0.001, **P < 0.01, *P < 0.05; ns, not significant. Between-group p-values are from the Wilcoxon rank-sum test (Cohort 1 vs Cohort 2; Cohort 1 vs Cohort 3). Cohort 1, IS-naïve + Telitacicept. Cohort 2, IS-naïve + SOC. Cohort 3, Refractory+Telitacicept. Complete withdrawal, glucocorticoid dose = 0 mg/d at Week 48.

### 3.4 Safety outcomes

The overall incidence of TEAEs was 50.0% in Cohort 1, 40.9% in Cohort 2, and 29.4% in Cohort 3. Upper respiratory tract infections were more frequent in Cohort 3 (17.6%) and Cohort 1 (12.5%). Conversely, conditions such as dermatitis (9.1%), herpes zoster (9.1%), and pneumonia (9.1%) were more prevalent in Cohort 2. All TEAEs were classified as mild to moderate (Grade 1–2), and no Grade 3 or higher events were observed in any cohort (**Table 4**).

**Table 4:** Treatment-emergent adverse events.

TEAE	Any Grade, n (%)			Grade ≥3, n (%)		
	Cohort 1 (N = 16)	Cohort 2 (N = 22)	Cohort 3 (N = 17)	Cohort 1 (N = 16)	Cohort 2 (N = 22)	Cohort 3 (N = 17)
Any TEAE	8 (50.0%)	9 (40.9%)	5 (29.4%)	0 (0.0%)	0 (0.0%)	0 (0.0%)
Upper respiratory tract infection	2 (12.5%)	1 (4.5%)	3 (17.6%)	0 (0.0%)	0 (0.0%)	0 (0.0%)
Urinary tract infection	2 (12.5%)	1 (4.5%)	0 (0.0%)	0 (0.0%)	0 (0.0%)	0 (0.0%)
Bacterial folliculitis	1 (6.2%)	0 (0.0%)	0 (0.0%)	0 (0.0%)	0 (0.0%)	0 (0.0%)
Dermatitis	1 (6.2%)	2 (9.1%)	0 (0.0%)	0 (0.0%)	0 (0.0%)	0 (0.0%)
Herpes zoster	1 (6.2%)	2 (9.1%)	0 (0.0%)	0 (0.0%)	0 (0.0%)	0 (0.0%)
Pneumonia	1 (6.2%)	2 (9.1%)	0 (0.0%)	0 (0.0%)	0 (0.0%)	0 (0.0%)
Allergic conjunctivitis	0 (0.0%)	0 (0.0%)	1 (5.9%)	0 (0.0%)	0 (0.0%)	0 (0.0%)
Otitis externa	0 (0.0%)	0 (0.0%)	1 (5.9%)	0 (0.0%)	0 (0.0%)	0 (0.0%)
Vaginitis	0 (0.0%)	1 (4.5%)	0 (0.0%)	0 (0.0%)	0 (0.0%)	0 (0.0%)

TEAE, treatment-emergent adverse event. Cohort 1, IS-naïve + Telitacicept. Cohort 2, IS-naïve + SOC. Cohort 3, Refractory+Telitacicept.

## 4 Discussion

This real-world, triple-cohort study provided valuable evidence of the effectiveness and safety of telitacicept for lupus nephritis, suggesting significant clinical benefits in both IS-naïve and refractory cohorts, aligning with previous studies (10), and showing a favorable advantage over existing biologic therapies across disease status (13).

In the IS-naïve setting, telitacicept showed numerically faster CR achievement and a favorable HR for time to CR compared with the SOC cohort. In addition, telitacicept for treating IS-naïve LN also showed higher CR and TR rates than the refractory cohort. These findings suggest a potential clinical benefit of using telitacicept earlier, i.e., in IS-naïve LN patients. Cohort 1 showed favorable improvements in renal function, proteinuria, hematological profile, and serum albumin compared with Cohorts 2 and 3, and these differences were statistically significant.

The novel dual-target mechanism of telitacicept, which simultaneously inhibits both the BAFF and APRIL pathways, may explain its strong treatment effects observed in our study (8, 9). By targeting a wider range of B-lineage cells, including plasma cells, telitacicept offers more complete suppression of humoral immunity than single-target agents, as evidenced by significant increases in complement levels and decreases in immunoglobulin levels in both IS-naïve and refractory cohorts.

In this study, telitacicept demonstrated a favorable safety profile for treating both IS-naïve and refractory LN, with adverse events primarily mild-to-moderate infections and no Grade 3 or higher events. The higher incidence of upper respiratory tract infections in Cohort 3 and Cohort 1 is consistent with the known infectious risk associated with B-cell modulation, especially in those with prior immunosuppressive exposure. The higher prevalence of dermatitis, herpes zoster, and pneumonia in Cohort 2 may reflect prolonged or higher-dose glucocorticoid exposure in patients receiving SOC therapy alone. The infection profile was manageable and aligned with the background immunosuppression in LN. These observations, together with the glucocorticoid-sparing effect observed in this study, support telitacicept’s potential to reduce steroid-related complications while maintaining disease control (14–17). The complete withdrawal rates for steroids, particularly the higher rate observed in the refractory cohort, should be interpreted cautiously in this real-world, EMR-based setting. In the absence of protocol-driven tapering schedules, the reasons for discontinuation, including physician preference, drug resistance, or adverse events, could not be systematically ascertained. Therefore, while these findings are encouraging, they should be considered hypothesis-generating rather than confirmatory evidence of steroid-free remission.

Several limitations warrant consideration. The retrospective design and reliance on electronic medical records (EMR) may introduce inherent biases. In particular, EMR-derived safety data may underreport asymptomatic laboratory abnormalities, and the absence of standardized safety assessments limits systematic grading. The small sample size limited statistical power, which may explain the non-significant findings despite consistent numerical trends. The absence of a contemporaneous control group and repeat renal biopsies limits interpretability. The bicentric design may limit generalizability. Regarding refractory disease, all 17 patients met the 2019 EULAR/ERA-EDTA criteria for inadequate response, though 4 of 17 (23.5%) had failed only a single prior regimen, reflecting real-world practice in China (10), and differed from recent 2024 American College of Rheumatology (ACR) guideline defines refractory disease as failure to achieve at least a partial renal response after at least two different appropriate 6-month courses of immunosuppressive therapy (18). The significantly higher Chronicity Index in the refractory cohort further supports the more advanced disease status at baseline in these patients, which may contribute to the observed differences in treatment response. Furthermore, because the electronic medical record data were not originally intended for research, we could not systematically assess renal relapse status. This limitation precluded us from interpreting the long-term durability of treatment responses in this study. Despite these limitations, the consistency of our findings with prior telitacicept real-world studies (10) supports the clinical utility of telitacicept across disease status and underscores the strategic importance of early targeted therapy in LN management (19). Our results should be considered hypothesis-generating and warrant confirmation in larger prospective trials.

## 5 Conclusion

In conclusion, telitacicept is an effective and safe adjunctive therapy for lupus nephritis, showing a favorable renal response in both IS-naïve and refractory patients. The trend toward faster CR achievement with telitacicept in IS-naïve and refractory LN patients, along with a higher response rate in the refractory cohort, suggests a potential benefit of early intervention, including a glucocorticoid-sparing effect and manageable safety. These real-world findings support the early use of telitacicept in clinical practice and emphasize the need for larger, randomized controlled trials to confirm the benefits of early dual-target B-cell therapy in LN.

## Data Availability

The raw data supporting the conclusions of this article will be made available by the authors, without undue reservation.
