# Supplementary material for: A real-world, triple-cohort study on the effectiveness and safety of telitacicept in immunosuppressant-naïve and refractory lupus nephritis
Source: Front Immunol. 2026 Sep 16;17:1748931. doi: 10.3389/fimmu.2026.1748931 (PMC13623623; doi:10.3389/fimmu.2026.1748931)
Supplement: Supplementary file 1 [file Table1.docx]

Table A1. Prior Immunosuppressive (IS) Exposure in the Refractory Cohort (N=17)

| Prior Therapy | n (%) | Median Duration (IQR), years |
| --- | --- | --- |
| Corticosteroids | 17 (100) | 3.0 (1.5-5.0) |
| Mycophenolate mofetil (MMF) | 15 (88.2) | 2.0 (1.0-4.0) |
| Cyclophosphamide (CTX) | 6 (35.3) | 1.0 (1.0-2.0) |
| Tacrolimus | 7 (41.2) | 1.0 (0.5-2.0) |
| Leflunomide | 4 (23.5) | 2.0 (1.0-2.5) |
| Methotrexate | 2 (11.8) | 3.0 (2.0-4.0) |
| Cyclosporine | 1 (5.9) | 1.0 (1.0-1.0) |
| Thalidomide | 1 (5.9) | 1.0 (1.0-1.0) |
| Imatinib | 1 (5.9) | 0.5 (0.5-0.5) |
| Combination of prior IS agents |  |  |
| ≥ 2 different IS agents | 13 (88.2) | — |
| ≥ 3 different IS agents | 10 (58.8) | — |
| Prednisolone + CTX | 1 (5.9) | 2 |
| Prednisolone + MMF | 3 (17.6) | 3.0 (1.0-5.0) |
