# Supplementary material for: A real-world, triple-cohort study on the effectiveness and safety of telitacicept in immunosuppressant-naïve and refractory lupus nephritis
Source: Front Immunol. 2026 Sep 16;17:1748931. doi: 10.3389/fimmu.2026.1748931 (PMC13623623; doi:10.3389/fimmu.2026.1748931)
Supplement: Supplementary file 2 [file Table2.docx]

Table A2. Immunosuppressive Regimens

| Immunosuppressants | Cohort 1  n/N (%) | Cohort 2  n/N (%) | Cohort 3  n/N (%) |
| --- | --- | --- | --- |
| MMF | 16/16 (100.0%) | 13/22 (59.1%) | 15/17 (88.2%) |
| Cyclophosphamide | 0/16 (0%) | 1/22 (4.5%) | 1/17 (5.9%) |
| Tacrolimus | 0/16 (0%) | 2/22 (9.1%) | 0/17 (0%) |
| Leflunomide | 0/16 (0%) | 3/22 (13.6%) | 0/17 (0%) |
| MMF + Tacrolimus | 0/16 (0%) | 2/22 (9.1%) | 1/17 (5.9%) |
| Steroid only | 0/16 (0%) | 1/22 (4.5%) | 0/17 (0%) |
| SOC, standard of care; MMF, mycophenolate mofetil; TAC, tacrolimus. Cohort 1, IS-naïve + Telitacicept. Cohort 2, IS-naïve + SOC. Cohort 3, Refractory+Telitacicept. Glucocorticoid: the dose had been standardized to methylprednisolone. Cohort 2, IS-naïve +SOC. Cohort 3, Refractory+Telitacicept. | | | |
