# Supplementary material for: A real-world, triple-cohort study on the effectiveness and safety of telitacicept in immunosuppressant-naïve and refractory lupus nephritis
Source: Front Immunol. 2026 Sep 16;17:1748931. doi: 10.3389/fimmu.2026.1748931 (PMC13623623; doi:10.3389/fimmu.2026.1748931)
Supplement: Supplementary file 3 [file Table3.docx]

Table A3. Complete baseline characteristics

| Characteristic | A. Cohort 1  (N=16) | B. Cohort 2  (N=22) | C. Cohort 3  (N=17) | p Values | |
| --- | --- | --- | --- | --- | --- |
|  |  |  |  | A vs B | A vs C |
| Gender, n (%) |  |  |  | 0.234 | 1.000 |
| Female | 14 (87.5) | 16 (72.7) | 15 (88.2) |  |  |
| Male | 2 (12.5) | 6 (27.3) | 2 (11.8) |  |  |
| Age (years) | 31.9 ± 9.4 | 42.7 ± 21.0 | 37.0 ± 12.1 | 0.085 | 0.367 |
| Activity Index, median (IQR) | 10 (6.8, 11) | 6 (5, 10.8) | 10 (9, 12) | 0.136 | 0.675 |
| Chronicity Index, median (IQR) | 1 (1, 2.2) | 1 (0, 2) | 3 (2, 3) | 0.459 | 0.029 |
| SLEDAI-2K score, median (IQR) | 13 (10, 17) | 10 (10, 12) | 10 (10, 14) | 0.042 | 0.263 |
| eGFR (mL/min/1.73m²) | 86.2 ± 42.5 | 88.0 ± 48.4 | 58.5 ± 35.2 | 0.916 | 0.050 |
| 24h urine protein (g/d) | 4.8 ± 2.2 | 3.6 ± 3.1 | 3.4 ± 3.3 | 0.192 | 0.081 |
| UPCR (mg/mmol) | 445.4 ± 269.0 | 428.7 ± 282.6 | 498.5 ± 580.5 | 0.856 | 0.460 |
| ESR (mm/h) | 65.2 ± 32.5 | 65.3 ± 37.8 | 43.7 ± 37.5 | 0.849 | 0.058 |
| IgA (g/L) | 3.25 ± 0.87 | 2.76 ± 1.07 | 2.15 ± 0.92 | 0.147 | 0.004 |
| IgM (g/L) | 1.26 ± 0.8 | 1.32 ± 0.77 | 1.13 ± 0.94 | 0.701 | 0.288 |
| IgG (g/L) | 10.58 ± 5.39 | 11.29 ± 5.08 | 11.11 ± 4.52 | 0.929 | 0.957 |
| Complement C3 (g/L) | 0.38 ± 0.27 | 0.49 ± 0.20 | 0.58 ± 0.31 | 0.185 | 0.050 |
| Complement C4 (g/L) | 0.07 ± 0.06 | 0.11 ± 0.06 | 0.14 ± 0.10 | 0.066 | 0.020 |
| Glucocorticoid (mg/d) | 37.6 ± 12.5 | 42.3 ± 8.8 | 19.2 ± 16.5 | 0.184 | 0.005 |
| Biopsy class, n (%) |  |  |  | 0.490 | 0.162 |
| Class III | 0 (0%) | 1 (4.5%) | 3 (17.6%) |  |  |
| Class IV | 5 (31.2%) | 4 (18.2%) | 4 (23.5%) |  |  |
| Class V | 3 (18.8%) | 6 (27.3%) | 0 (0%) |  |  |
| Class III/V | 2 (12.5%) | 6 (27.3%) | 3 (17.6%) |  |  |
| Class IV/V | 6 (37.5%) | 4 (18.2%) | 5 (29.4%) |  |  |
| Class III/IV | 0 (0%) | 0 (0%) | 0 (0%) |  |  |
| Autoantibody-positive |  |  |  |  |  |
| ANA | 16/16 (100%) | 20/22 (90.9%) | 16/17 (94.1%) | 0.499 | 1.000 |
| Anti-dsDNA | 6/16 (37.5%) | 7/22 (31.8%) | 6/17 (35.3%) | 0.742 | 1.000 |
| Anti-nucleosome | 9/16 (56.2%) | 6/22 (27.3%) | 10/17 (58.8%) | 0.099 | 1.000 |
| Anti-histone | 6/16 (37.5%) | 1/22 (4.5%) | 8/17 (47.1%) | 0.028 | 0.728 |
| Anti-SSA/Ro | 8/16 (50.0%) | 15/22 (68.2%) | 5/17 (29.4%) | 0.324 | 0.296 |
| Anti-RNP/Sm | 5/16 (31.2%) | 8/22 (36.4%) | 7/17 (41.2%) | 1.000 | 0.721 |
| Anti-Ro-52 | 7/16 (43.8%) | 13/22 (59.1%) | 4/17 (23.5%) | 0.512 | 0.282 |
| Anti-ribosomal P | 4/16 (25.0%) | 8/22 (36.4%) | 7/17 (41.2%) | 0.504 | 0.465 |
| Anti-Sm | 4/16 (25.0%) | 8/22 (36.4%) | 5/17 (29.4%) | 0.504 | 1.000 |
| Anti-SSB/La | 2/16 (12.5%) | 2/22 (9.1%) | 1/17 (5.9%) | 1.000 | 0.601 |
| Glucocorticoid (mg/d) | 37.6 ± 12.5 | 42.3 ± 8.8 | 19.2 ± 16.5 | 0.184 | 0.005 |
| Data were presented as mean ± SD unless otherwise specified. *p value for Cohort 1 vs Cohort 2; †p value for Cohort 1 vs Cohort 3. UP24h: 24-hour urine protein; GC: glucocorticoids; eGFR: estimated glomerular filtration rate; Anti-dsDNA: anti-double-stranded DNA; SLEDAI-2K: Systemic Lupus Erythematosus Disease Activity Index 2000; MMF: mycophenolate mofetil; IQR: interquartile range. Cohort 1, IS-naïve + Telitacicept. Cohort 2, IS-naïve + SOC. Cohort 3, Refractory+Telitacicept. Glucocorticoid: the dose had been standardized to methylprednisolone. Cohort 2, IS-naïve +SOC. Cohort 3, Refractory+Telitacicept. | | | | | |
