# Supplementary material for: A real-world, triple-cohort study on the effectiveness and safety of telitacicept in immunosuppressant-naïve and refractory lupus nephritis
Source: Front Immunol. 2026 Sep 16;17:1748931. doi: 10.3389/fimmu.2026.1748931 (PMC13623623; doi:10.3389/fimmu.2026.1748931)
Supplement: Supplementary file 4 [file Table4.docx]

Table A4. LSM Changes in Laboratory Parameters from Baseline to Week 24

| Measures | LSM change from baseline (95% CI) ^†^ | | |  | p value | |
| --- | --- | --- | --- | --- | --- | --- |
|  | A. Cohort 1  (N=16) | B. Cohort 2  (N=22) | C. Cohort 3  (n=17) |  | A vs B | A vs C |
| eGFR (mL/min/1.73m²) | 19.0  (8.6, 29.4) ^***^ | 8.6  (-0.7, 17.9) ^ns^ | 6.8  (-3.9, 17.6) ^ns^ |  | 0.140 | 0.110 |
| UPCR (mg/mmol) | -368.69  (-447.33, -290.05) ^****^ | -341.60  (-408.77, -274.43) ^****^ | -281.51  (-357.98, -205.04) ^****^ |  | 0.600 | 0.120 |
| C3 (g/L) | 0.42  (0.33, 0.51) ^****^ | 0.26  (0.19, 0.34) ^****^ | 0.29  (0.2, 0.37) ^****^ |  | 0.010 | 0.050 |
| C4 (g/L) | 0.10  (0.08, 0.13) ^****^ | 0.08  (0.05, 0.1) ^****^ | 0.09  (0.07, 0.12) ^****^ |  | 0.160 | 0.610 |
| IgA (g/L) | -1.56  (-2.02, -1.1) ^****^ | -0.57  (-0.94, -0.19) ^**^ | -0.98  (-1.43, -0.53) ^****^ |  | 0.001 | 0.090 |
| IgG (g/L) | -2.76  (-4.62, -0.9) ^**^ | -2.04  (-3.63, -0.46) ^*^ | -1.88  (-3.68, -0.08) ^*^ |  | 0.560 | 0.500 |
| IgM (g/L) | -0.65  (-0.89, -0.41) ^****^ | -0.33  (-0.54, -0.13) ^**^ | -0.65  (-0.88, -0.41) ^**^ |  | 0.050 | 0.980 |
| Serum Albumin (g/L) | 13.84  (11.7, 15.97) ^****^ | 9.69  (7.89, 11.49) ^****^ | 11.68  (9.58, 13.78) ^****^ |  | 0.004 | 0.160 |
| Hb (g/L) | 20.12  (14.46, 25.79) ^****^ | 13.92  (9.02, 18.82) ^****^ | 12.64  (7.14, 18.13) ^****^ |  | 0.110 | 0.060 |
| BPC (×10⁹/L) | 49.37  (22.38, 76.35) ^***^ | 40.73  (18.17, 63.29) ^***^ | 54.13  (28.05, 80.2) ^***^ |  | 0.620 | 0.800 |
| LSM, least squares mean. C3, C4, complement C3, C4. Ig, immunoglobulin. BPC, blood platelet count. † Indicated p-values within the cohort for LSM changes in measures from baseline: **** P < 0.0001, *** P < 0.001, ** P < 0.01, * P < 0.05, ns, not significant. Cohort 1, IS-naïve + Telitacicept. Cohort 2, IS-naïve +SOC. Cohort 3, Refractory+Telitacicept. | | | | | | |
